# *Cryptosporidium* Species and *C. parvum* Subtypes in Farmed Bamboo Rats

**DOI:** 10.3390/pathogens9121018

**Published:** 2020-12-02

**Authors:** Falei Li, Wentao Zhao, Chenyuan Zhang, Yaqiong Guo, Na Li, Lihua Xiao, Yaoyu Feng

**Affiliations:** 1 Center for Emerging and Zoonotic Diseases, South China Agricultural University, Wushan Road, Guangzhou 510642, China; falei0316@126.com (F.L.); 17818522213@163.com (W.Z.); chenyuanzhang@gdaib.edu.cn (C.Z.); guoyq@scau.edu.cn (Y.G.); nli@scau.edu.cn (N.L.); 2 Guangdong Laboratory for Lingnan Modern Agriculture, Wushan Road, Guangzhou 510642, China

**Keywords:** *Cryptosporidium parvum*, subtype, bamboo rat, human pathogen

## Abstract

Bamboo rats (*Rhizomys sinensis*) are widely farmed in Guangdong, China, but the distribution and public health potential of *Cryptosporidium* spp. in them are unclear. In this study, 724 fecal specimens were collected from bamboo rats in Guangdong Province and analyzed for *Cryptosporidium* spp. using PCR and sequence analyses of the small subunit rRNA gene. The overall detection rate of *Cryptosporidium* spp. was 12.2% (88/724). By age, the detection rate in animals under 2 months (23.2% or 13/56) was significantly higher than in animals over 2 months (11.2% or 75/668; χ^2^ = 6.95, *df* = 1, *p* = 0.0084). By reproduction status, the detection rate of *Cryptosporidium* spp. in nursing animals (23.1% or 27/117) was significantly higher than in other reproduction statuses (6.8% or 4/59; χ^2^ = 7.18, *df* = 1, *p* = 0.0074). Five *Cryptosporidium* species and genotypes were detected, including *Cryptosporidium* bamboo rat genotype I (*n* = 49), *C. parvum* (*n* = 31), *Cryptosporidium* bamboo rat genotype III (*n* = 5), *C. occultus* (*n* = 2), and *C. muris* (*n* = 1). The average numbers of oocysts per gram of feces for these *Cryptosporidium* spp. were 14,074, 494,636, 9239, 394, and 323, respectively. The genetic uniqueness of bamboo rat genotypes I and III was confirmed by sequence analyses of the 70 kDa heat shock protein and actin genes. Subtyping *C. parvum* by sequence analysis of the 60 kDa glycoprotein gene identified the presence of IIoA15G1 (*n* = 20) and IIpA6 (*n* = 2) subtypes. The results of this study indicated that *Cryptosporidium* spp. are common in bamboo rats in Guangdong, and some of the *Cryptosporidium* spp. in these animals are known human pathogens.

## 1. Introduction

*Cryptosporidium* spp. are major pathogens that mainly parasitize the gastrointestinal epithelium, causing moderate-to-severe diarrhea in humans and animals [1,2]. They are responsible for significant mortality in both young children [1,3] and neonatal farm animals [4,5,6,7,8]. In addition, cryptosporidiosis has been associated with retarded growth in humans and farm animals [9,10,11,12].

*Cryptosporidium* spp. are especially common in rodents [13]. Among the >40 established *Cryptosporidium* species and an equal number of genotypes of unknown species status [14], *C. parvum* is commonly found in various rodents in China [15]. Other *Cryptosporidium* spp. from rodents have more limited host ranges, such as *C. meleagridis* and *Cryptosporidium* deer mouse genotypes I, II, III, and IV in deer mice [16,17,18,19]; *Cryptosporidium* chipmunk genotypes I and II in chipmunks [16]; and *Cryptosporidium* ferret genotype, *C. rubeyi* and several squirrel genotypes in squirrels [20,21,22,23].

Bamboo rats are widely farmed in southern China. They are commonly infected with *Cryptosporidium* spp. Several *Cryptosporidium* species and genotypes have been identified in these animals, including *C. parvum*, *C. parvum*-like genotype, *C. occultus*, and *Cryptosporidium* bamboo rat genotypes I and II [24,25]. Thus, the distribution of *Cryptosporidium* spp. in bamboo rats appears to be different from other rodents. At the subtype level, *C. parvum* subtypes found in bamboo rats also differ from those in other rodents. In China, rodents such as hamsters, chipmunks, and rats are mostly infected with the IId subtype family of *C. parvum*, with IIdA15G1 and IIdA19G1 as the most common subtypes [26]. In contrast, bamboo rats in southern China are seemingly infected with rare IIo and IIp subtype families of *C. parvum* [25,27]. Most of data on these two *C. parvum* subtype families in bamboo rats, however, were from a study of animals in Jiangxi, Guangxi, and Hainan [24].

In this study, we examined the occurrence of *Cryptosporidium* spp. and *C. parvum* subtypes in bamboo rats in Guangdong Province. The distributions of *Cryptosporidium* spp. and *C. parvum* subtypes were compared among farms, age groups and reproduction statuses. The oocyst shedding intensity was compared among *Cryptosporidium* species and genotypes for the first time.

## 2. Results

### 2.1. Occurrence of Cryptosporidium spp. in Bamboo Rats

The overall detection rate of *Cryptosporidium* spp. in bamboo rats in Guangdong was 12.2% (88/724). The detection rate of *Cryptosporidium* spp. on farm 1 (35.9% or 33/92) was significantly higher than on farm 2 (3.5% or 5/142; χ^2^ = 42.95, *df* = 1, *p* < 0.0001; *df*: degrees of freedom), farm 3 (1.0% or 2/205; χ^2^ = 74.38, *df* = 1, *p* < 0.0001), farm 5 (0% or 0/56; χ^2^ = 25.85, *df* = 1, *p* < 0.0001), and farm 6 (19.7% or 37/188; χ^2^ = 8.63, *df* = 1, *p* = 0.0033). The difference in detection rate between farms 4 (26.8% or 11/41) and 1 was not significant (χ^2^ = 1.05, *df* = 1, *p* = 0.3062; Table 1).

By age, the detection rates of *Cryptosporidium* spp. in bamboo rats ranged from 0.0% (0/8) in animals of 6–8 months to 23.2% (13/56) in animals under 2 months. The detection rate of *Cryptosporidium* spp. in animals under 2 months of age was significantly higher than in older animals (11.2% or 75/668; χ^2^ = 6.95, *df* = 1, *P* = 0.0084; Table 2). By reproduction status of adult animals (1–3 years in age), nursing animals had a significantly higher detection rate (23.1% or 27/117) than breeding animals (4.9% or 10/205; χ^2^ = 24.26, *df* = 1, *p* < 0.0001), pregnant animals (6.8% or 4/59; χ^2^ = 7.18, *df* = 1, *p* = 0.0074), and nonpregnant animals (10.6% or 21/198; χ^2^ = 8.86, *df* = 1, *p* = 0.0029; Table 3).

### 2.2. Cryptosporidium Species and Genotypes Identified

**Figure 1:**
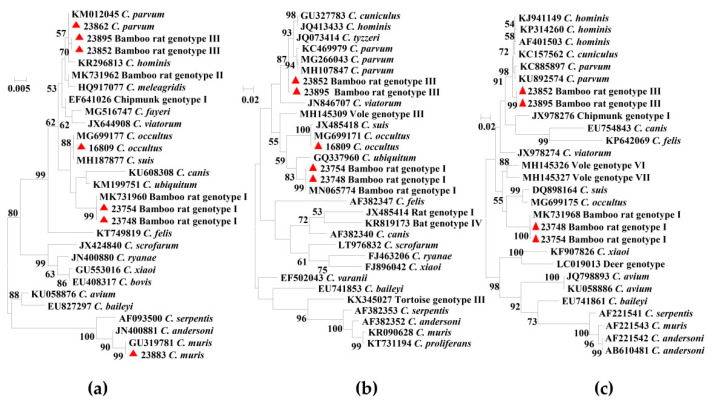
Phylogenetic relationship of *Cryptosporidium* spp. based on the maximum likelihood analyses of the *SSU* rRNA gene (**a**); actin gene (**b**) and 70 kDa heat shock protein gene (**c**). Bootstrap values greater than 50% from 1000 replicates are displayed. The red triangles indicate the *Cryptosporidium* spp. identified in the present study.

All 88 *Cryptosporidium*-positive specimens were successfully genotyped by sequence analysis of the *SSU* rRNA gene. Among them, five *Cryptosporidium* species and genotypes were detected, including bamboo rat genotype I in 49 specimens, *C. parvum* in 31 specimens, bamboo rat genotype III in 5 specimens, *C. occultus* in 2 specimens, and *C. muris* in 1 specimen. The *SSU* rRNA sequences from bamboo rat genotype I, bamboo rat genotype III, *C. parvum*, and *C. occultus* were identical to reference sequences MK956935 (from bamboo rats in China), MK956936 (from bamboo rats in China), MK956932 (from bamboo rats in China), and MK982467 (from calves in Bangladesh), respectively. The bamboo rat genotype III was previously named the *C. parvum*-like genotype. In contrast, the sequence from *C. muris* was similar to the reference sequence GU319781 from nonhuman primates in China, with three nucleotide substitutions. In phylogenetic analysis of the *SSU* rRNA sequences, these *Cryptosporidium* species and genotypes from bamboo rats clustered with their reference sequences (Figure 1a).

Representative isolates of bamboo rat genotypes I and III and *C. occultus* were characterized by sequence analysis of the actin gene. The sequences from bamboo rat genotype I and *C. occultus* were identical to reference sequences MN065774 (from bamboo rats in China) and MG699171 (from *Meriones unguiculatus* in Czech Republic), respectively. The sequence from bamboo rat genotype III, however, was similar to the *C. parvum* sequence MG266043 from *Apodemus agrarius* in Slovakia, with 16 nucleotide substitutions. As expected, these *Cryptosporidium* species and genotypes clustered with their reference sequences in phylogenetic analysis of the actin gene (Figure 1b).

Nucleotide sequences of the *hsp70* gene were also obtained from representative isolates of bamboo rat genotypes I and III; the two *C. occultus* isolates were PCR-negative at this locus. The sequences from bamboo rat genotype I was identical to reference sequences MK731968 from bamboo rats in China. In contrast, the sequences from bamboo rat genotype III had 14 single nucleotide polymorphisms (SNPs) and 1 nucleotide deletion compared with *C. parvum* sequences (AF401503 and KU892574). As expected, bamboo rat genotypes I and III clustered these reference sequences in phylogenetic analysis of the *hsp70* gene (Figure 1c).

### 2.3. Age Pattern of Cryptosporidium Species and Genotypes

Bamboo rat genotype I and *C. parvum* were the dominant *Cryptosporidium* spp. in bamboo rats, especially in animals under 2 months of age and in nursing adults. Among adult animals, both *Cryptosporidium* spp. were detected in nursing animals and breeding animals. In contrast, only bamboo rat genotype I was detected in pregnant and nonpregnant animals (Table 3). Among young animals, both bamboo rat genotype I and *C. parvum* were detected in animals under 4 months of age. They, however, were not detected in animals with an age of 4–8 months (Table 2).

### 2.4. Occurrence of C. parvum Subtypes

Twenty-two of the 31 *C. parvum*-positive specimens were successfully subtyped based on sequence analysis of the *gp60* gene. Subtypes IIoA15G1 (*n* = 20) and IIpA6 (*n* = 2) were identified from them. The sequences generated were identical to reference sequences MK956002 and MK955996, respectively.

### 2.5. Intensity of Oocyst Shedding among Cryptosporidium spp.

**Figure 2:**
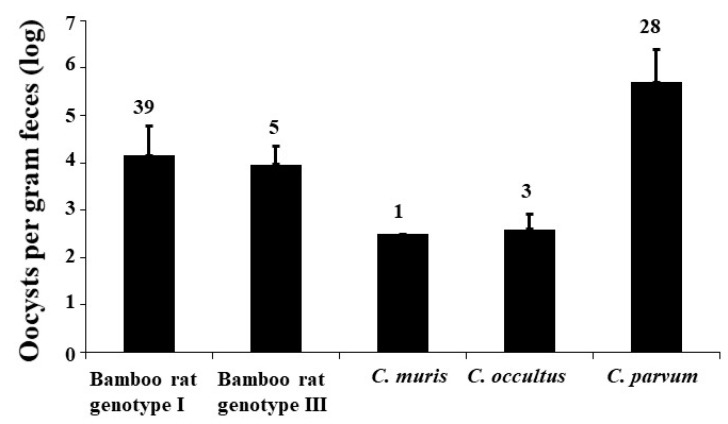
Oocyst shedding intensity (oocysts per gram of feces) of *Cryptosporidium* species and genotypes in bamboo rats (mean ± standard deviation).

The oocyst shedding intensity, oocysts per gram of feces (OPG), in *Cryptosporidium*-infected bamboo rats was measured using qPCR. The average OPG values were 494,636 ± 1,892,289 for *C. parvum* (*n* = 28), 9239 ± 12,939 for bamboo rat genotype III (*n* = 5), 14,074 ± 46,621 for bamboo rat genotype I (*n* = 39), 394 ± 442 for *C. occultus* (*n* = 3), and 323 for *C. muris* (*n* = 1; Figure 2).

## 3. Discussion

The results of this study suggest that *Cryptosporidium* spp. are common in bamboo rats in Guangdong, China. The detection rate of *Cryptosporidium* spp. in present study (12.2% or 88/724) is higher than in previous study (2.2% or 1/46) in the same area [25]. It is also higher than the 3.2% detection rate in bamboo rats from a pet market [27]. In contrast, it is lower than several reports of *Cryptosporidium* spp. in farmed bamboo rats sampled in Guangxi (20.9% or 100/477), Jiangxi (33.3% or 51/153), and Hainan (69.6% or 55/79) [24]. Among six farms, the detection rate of *Cryptosporidium* spp. on farm 1 (35.9%) was higher than other farms (0.0–26.8%), probably because of the higher animal density and poor hygiene conditions on the farm. The detection rates of *Cryptosporidium* spp. in animals under 2 months of age (23.2%) and nursing animals (23.1%) were higher than other age groups (0.0–19.4%) and reproduction statuses (4.9–10.6%). These differences might be due to the lower immunity of young animals and nursing animals, making them more susceptible to infection with *Cryptosporidium* spp. As the detection rates differed among age groups and reproduction statuses, these variations in detection rates among studies are expected.

Diverse *Cryptosporidium* species and genotypes are present in farmed bamboo rats in the study area. Five *Cryptosporidium* species and genotypes were identified in this study, including bamboo rat genotype I (55.7%, 49/88), *C. parvum* (35.2%, 31/88), bamboo rat genotype III (5.7%, 5/88), *C. occultus* (2.3%, 2/88), and *C. muris* (1.1%, 1/88). Among them, bamboo rat genotype I and *C. parvum* are dominant *Cryptosporidium* spp. in present study. This is similar to observations of two previous studies [24,25]. However, bamboo rat genotype III, commonly found in bamboo rats in Guangxi and Hainan [24], is rare in the present study. Among the five *Cryptosporidium* species and genotypes identified in the study, *C. parvum* is a well-known human pathogen and has been found in a wide range of animals. *Cryptosporidium muris* and *C. occultus*, in contrast, are mostly *Cryptosporidium* species of rats and have been only occasionally found in humans [14]. Although bamboo rat genotypes I and III are genetically related to *C. ubiquitum* and *C. parvum*, respectively, in sequence analysis of three genetic loci, it is still unclear whether they can infect humans [14,28].

Results of oocyst shedding intensity in this study suggest that bamboo rats could be natural hosts of bamboo rat genotype I, bamboo rat genotype III, and *C. parvum*. Among five *Cryptosporidium* spp. identified in this study, these three *Cryptosporidium* genotypes had higher oocyst shedding intensity than *C. occultus* and *C. muris*. In addition, numerous bamboo rats are known to be infected with bamboo rat genotypes I and III and *C. parvum*. In contrast, brown rats appear to be natural hosts of *C. occultus* and *C. muris* [29,30,31]. Infection with these two *Cryptosporidium* species in bamboo rats could be due to contact with *Cryptosporidium*-infected wild rats living in the same ecological niche.

The unique *C. parvum* subtypes identified in the study probably represent emerging pathogens in a broad range of hosts. Thus far, more than 20 *C. parvum* subtype families have been identified based on sequence analyses of the *gp*60 gene [32]. Among them, the IIo and IIp were previously seen in a few bamboo rats in Sichuan, China and were considered rare *C. parvum* subtype families [27]. IIo subtypes, however, have been found in numerous bamboo rats from Jiangxi, Guangxi, and Hainan in southern China [24]. One IIo subtype, IIoA14, has also been widely found in crab-eating macaques in Hainan, China [33]. Human infections with IIo subtypes have been identified in Thailand and New Zealand [34,35,36]. IIp subtypes of *C. parvum* also appear to be common in bamboo rats, having been thus far found in Jiangxi, Sichuan, Guangxi, Guangdong, and Hainan [24,27]. As they are genetically related to IId and IIo subtypes, they could also have a broader host range and human-infective potential [33]. Interestingly, IIp subtypes were much more prevalent than IIo subtypes in the previous survey of *Cryptosporidium* spp. in bamboo rats in Jiangxi, Guangxi, and Hainan. In the present study in Guangdong, the IIoA15G1 subtype was more commonly detected than the IIpA6 subtype.

There appears to be some transmission of *C. parvum* between nursing bamboo rats and their babies. Most *C. parvum* infections in adult bamboo rats (18 of 19 *C. parvum*-positive) were detected in nursing mothers; only one of them was found in another adult bamboo rat. In contrast, all *C. parvum* infections in young bamboo rats (12 of 12 *C. parvum*-positive) were detected in animals under 4 months of age. The *C. parvum* infections in nursing animals were probably due to sharing cages between the nursing dams and their babies. This is supported by the result of subtype analysis, in which the IIoA15G1 subtype was commonly found in both nursing animals and young bamboo rats. This is also consistent with results of studies in sheep and cattle, in which *C. parvum* is mostly detected in pre-weaned animals [37,38,39,40]. As the number of animals examined in the present study is small, further studies are needed to support this hypothesis. In contrast, *Cryptosporidium* bamboo rat genotype I appeared to be transmitted differently from *C. parvum*, as it was commonly found in adult bamboo rats of all reproduction statuses.

## 4. Materials and Methods

### 4.1. Ethics Statement

All fecal specimens were collected from bamboo rats with the approval of the farmers. The animals were handled in compliance with the regulations of the Chinese Laboratory Animal Administration Act of 2017. The study protocol was approved by the Research Ethics Committee of the South China Agricultural University (approval no. 2019g001).

### 4.2. Specimens

A total of 724 fecal specimens were collected during March to May 2019 from bamboo rats on six farms in Guangdong Province, China. On these farms, animals were kept in cages of 60 × 60 × 60 cm. Animals under six months of age were mostly kept in groups of 10, while older animals (between 7 months and 3 years) were kept in groups of 3. For the former, four specimens were collected from fresh feces in different corners of the cage, while for the latter, only one fecal specimen was collected from the cage (Table 1). These fecal specimens were stored in 2.5% potassium dichromate at 4 °C before DNA extraction.

The bamboo rats examined were divided into five age groups, including <2 months, 2–4 months, 4–6 months, 6–8 months, and 1–3 years (Table 2). The adult animals (1–3 years-old) were further divided into four reproduction statuses: nursing (female bamboo rats feeding babies), breeding (one female and 1–2 males kept together for mating), pregnancy, and nonpregnancy (Table 3). On the studies farms, pregnant bamboo rats were moved to new cages for delivery after for delivery after cleaning of the cages without any further disinfection.

### 4.3. Detection, Genotyping and Subtyping of Cryptosporidium spp.

Fecal specimens were washed off potassium dichromate with distilled water by centrifugation at 2000× *g* for 10 min. Genomic DNA was extracted from 200 mg washed fecal material using the Fast DNA Spin Kit for Soil (MP Biomedical, Santa Ana, CA, USA) as described [41]. *Cryptosporidium* spp. in the extracted DNA were detected using a nested PCR assay targeting a ~830-bp fragment of the small subunit rRNA (*SSU*) rRNA gene [42]. The *C. parvum* identified was subtyped by sequence analysis of a ~800-bp fragment of the 60 kDa (*gp60*) gene [43]. Representative isolates of the bamboo rat genotypes I and III and *C. occultus* were further characterized by sequence analyses of the 70 kDa heat shock protein (*hsp70*) and actin genes [44,45].

### 4.4. Measurement of Oocyst Shedding Intensity

The intensity of oocyst shedding in *Cryptosporidium*-positive specimens from naturally infected bamboo rats was measured by quantitative PCR (qPCR) targeting the *SSU* rRNA gene [33,46]. The qPCR was conducted on a LightCycler 480 II (Roche, Indianapolis, IN, USA). To calculate oocysts per gram of feces (OPG), Cq (quantitation cycle) values generated were analyzed based on a standard curve constructed with DNA preparations from fecal specimens spiked with known numbers of oocysts of the IOWA (USA) strain of *C. parvum* (Waterborne, Inc., New Orleans, LA, USA).

### 4.5. Sequence Analysis

All secondary PCR products from *Cryptosporidium*-positive specimens were sequenced bi-directionally on an ABI 3730 Autosequencer (Applied Biosystems, Foster City, CA, USA) for determining *Cryptosporidium* spp. and *C. parvum* subtypes. The nucleotide sequences generated were assembled using ChromasPro 2.1.5.0 (http://technelysium. com. au/ChromasPro. html), edited using BioEdit 7.1.3.0 (http://www.mbio.ncsu.edu/BioEdit/bioedit.html), and aligned with reference sequences downloaded from GenBank using ClustalX 2.0.11 (http://clustal.org). Phylogenetic analysis of sequence generated was performed by constructing maximum likelihood trees using Mega 6.0 (http://www.megasoftware.net/) based on substitution rates calculated using the general time reversible model. The robustness of clade formation was assessed using bootstrapping with 1000 replicates. Representative sequences generated in this study were submitted to the GenBank under accession numbers MW092529-MW092535 and MW117315-MW117325.

### 4.6. Statistical Analysis

The occurrence rates of *Cryptosporidium* spp. in bamboo rats were compared among farms, age groups, and reproduction statuses using the chi-square test implemented in SPSS v.20.0 (IBM Corp., New York, NY, USA). Differences were considered significant at *p* ≤ 0.05.

## 5. Conclusions

The results of this study suggest that divergent *Cryptosporidium* spp., including *C. parvum*, bamboo rat genotypes I and III, *C. occultus* and *C. muris,* occur in farmed bamboo rats in Guangdong, China. The *C. parvum* identified belongs to the unique subtype families IIo and IIp. As most of the *Cryptosporidium* species and *C. parvum* subtypes are not commonly found in domestic animals, they could have wildlife origin and have maintained at high transmission intensity in some of the semi-domesticated exotic animals. Attention should be paid to their spillover to other farm animals as well as human populations.

## Figures and Tables

**Table 1:** Occurrence of *Cryptosporidium* spp. and *C. parvum* subtypes in bamboo rats in Guangdong, China.

Farm	Age Group	No. Specimens	No. Positive (%)	*Cryptosporidium* spp.					*C. parvum* Subtype
				Bamboo Rat Genotype I	Bamboo Rat Genotype III	*C. occultus*	*C. muris*	*C. parvum*	
1	<2 months	9	6 (66.7)	6	-	-	-	-	-
	1–3 years	83	27 (32.5)	27	-	-	-	-	-
	subtotal	92	33 (35.9)	33	-	-	-	-	-
2	4–6 months	1	0 (0.0)	-	-	-	-	-	-
	1–3 years	141	5 (3.5)	4	-	1	-	-	-
	subtotal	142	5 (3.5)	4	-	1	-	-	-
3	<2 months	36	0 (0.0)	-	-	-	-	-	-
	2–4 months	29	0 (0.0)	-	-	-	-	-	-
	4–6 months	18	1 (5.6)	-	-	1	-	-	-
	6–8 months	8	0 (0.0)	-	-	-	-	-	-
	1–3 years	114	1 (0.9)	-	-	-	-	1	-
	subtotal	205	2 (1.0)	-	-	1	-	1	-
4	2–4 months	16	6 (37.5)	3	-	-	-	3	IIoA15G1 (1)
	1–3 years	25	5 (20.0)	5	-	-	-	-	-
	subtotal	41	11 (26.8)	8	-	-	-	3	IIoA15G1 (1)
5	1–3 years	56	0 (0.0)	-	-	-	-	-	-
	subtotal	56	0 (0.0)	-	-	-	-	-	-
6	<2 months	11	7 (63.6)	-	-	-	-	7	IIoA15G1 (5)
	2–4 months	17	6 (35.3)	3	1	-	-	2	IIoA15G1 (1)
	1–3 years	160	24 (15.0)	1	4	-	1	18	IIoA15G1 (13), IIpA6 (2)
	subtotal	188	37 (19.7)	4	5	-	1	27	IIoA15G1 (19), IIpA6 (2)
	Total	724	88 (12.2)	49	5	2	1	31	IIoA15G1 (20), IIpA6 (2)

**Table 2:** Occurrence of *Cryptosporidium* spp. in farmed bamboo rats in Guangdong by age.

Age	No. Specimens	No. Positive (%)	*Cryptosporidium* spp.					*C. parvum* Subtype
			Bamboo Rat Genotype I	Bamboo Rat Genotype III	*C. muris*	*C. occultus*	*C. parvum*	
<2 months	56	13 (23.2)	6	-	-	-	7	IIoA15G1 (5)
2–4 months	62	12 (19.4)	6	1	-	-	5	IIoA15G1 (2)
4–6 months	19	1 (5.3)	-	-	-	1	-	-
6–8 months	8	0 (0.0)	-	-	-	-	-	-
1–3 years	579	62 (10.7)	37	4	1	1	19	IIoA15G1 (13), IIpA6 (2)
Total	724	88 (12.2)	49	5	1	2	31	IIoA15G1 (20), IIpA6 (2)

**Table 3:** Occurrence of *Cryptosporidium* spp. in farmed adult bamboo rats in Guangdong by reproduction status.

Animal Status	No. Specimens	No. Positive (%)	*Cryptosporidium* spp.					*C. parvum* Subtype
			Bamboo Rat Genotype I	Bamboo Rat Genotype III	*C. muris*	*C. occultus*	*C. parvum*	
Nursing	117	27 (23.1)	5	3	1	-	18	IIoA15G1(13), IIpA6 (2)
Breeding	205	10 (4.9)	8	1	-	-	1	-
Pregnancy	59	4 (6.8)	4	-	-	-	-	-
Nonpregnant	198	21 (10.6)	20	-	-	1	-	-
Total	579	62 (10.7)	37	4	1	1	19	IIoA15G1 (13), IIpA6 (2)
